# Supplementary material for: Breakdown of supersaturation barrier links protein folding to amyloid formation
Source: Commun Biol. 2021 Jan 26;4:120. doi: 10.1038/s42003-020-01641-6 (PMC7838177; doi:10.1038/s42003-020-01641-6)
Supplement: Supplementary file 2 — Supplementary Information [file 42003_2020_1641_MOESM2_ESM.pdf]

Supplementary Information for

Breakdown of supersaturation barrier links protein folding to amyloid formation

Masahiro Noji, Tatsushi Samejima, Keiichi Yamaguchi, Masatomo So, Keisuke Yuzu, Eri Chatani, Yoko Akazawa-Ogawa, Yoshihisa Hagihara, Yasushi Kawata, Kensuke Ikenaka, Hideki Mochizuki, József Kardos, Daniel E. Otzen, Vittorio Bellotti, Johannes Buchner, and Yuji Goto

Correspondence to: gtyj8126@protein.osaka-u.ac.jp

This PDF file includes:

Supplementary Table 1

Supplementary Figs. 1 to 13

Supplementary references

Other Supplementary Material for this manuscript include the following:

Supplementary Movie 1

Supplementary Data 1

Protein	Amino acid residues	Main chain ΔS	Exp. pH	CamSol ave. hydro-phobicity	CamSol score	Protein-Sol score	AGGRESCAN THSAr	Tango AGG/length	Charge at exp. pH	Transition types
K3	22	462	2.0	0.57	0.77	0.71	0.17	0.89	2.9	A
pOVA	23	483	8.0	0.87	0.09	0.71	0.35	40.13	-0.5	
Glucagon	29	609	7.0	0.37	1.18	0.45	0.09	1.19	0.2	
IAPP	37	777	7.0	0.29	1.16	0.63	0.16	1.38	2.1	
$A\beta_{40}$	40	840	7.0	0.53	0.66	0.77	0.27	21.92	-2.4	
Insulin	51		7.0						-1.8	
β2m	100	2079	7.0	0.32	0.92	0.59	0.11	7.84	-1.2	S
C_L	101	2226	7.0	0.42	0.48	0.74	0.13	3.46	-1.7	
V_L (PAT)	112	2352	7.0	0.44	0.26	0.62	0.16	4.27	-1.9	
Ubiquitin	76	1596	2.0	0.22	1.33	0.76	0.13	0.16	12.9	
αLA	123	2583	2.0	0.23	0.86	0.66	0.09	1.92	16.8	
RNaseA	124	2604	5.0	0.18	1.22	0.69	0.02	1.17	9.9	
TTR	127	2667	2.0	0.23	1.09	0.73	0.16	3.32	16.9	
HEWL	129	2709	2.0	0.11	1.54	0.54	0.07	0.44	18.9	
OVA	140	2940	7.0	0.20	1.64	1.00	0.09	5.68	-8.8	
αSyn	386	8106	8.0	0.33	-0.47	0.43	0.14	7.15	-14.0	B
TDP-43	414	8694	7.0	0.18	1.18	0.31	0.08	4.20	-3.0	
Tau	441	9261	7.0	0.02	3.08	0.59	0.01	0.10	4.8	

Supplementary Table 1. The possible solubility-related factors important for determining different aggregation types of proteins and peptides. CamSol hydrophobicity and solubility scores[1], protein solubility socres[2], AGGRESCAN scores[3], Tango AGG scores[3] and net charge values were estimated. Amino acid residues, main chain Δ$\boldsymbol{S}_{\mathbf{conf}}$, experimental pH and transition types are the same as Table 1.
The values for vacant cells of insulin were not estimated because of the complexity caused by two chains.

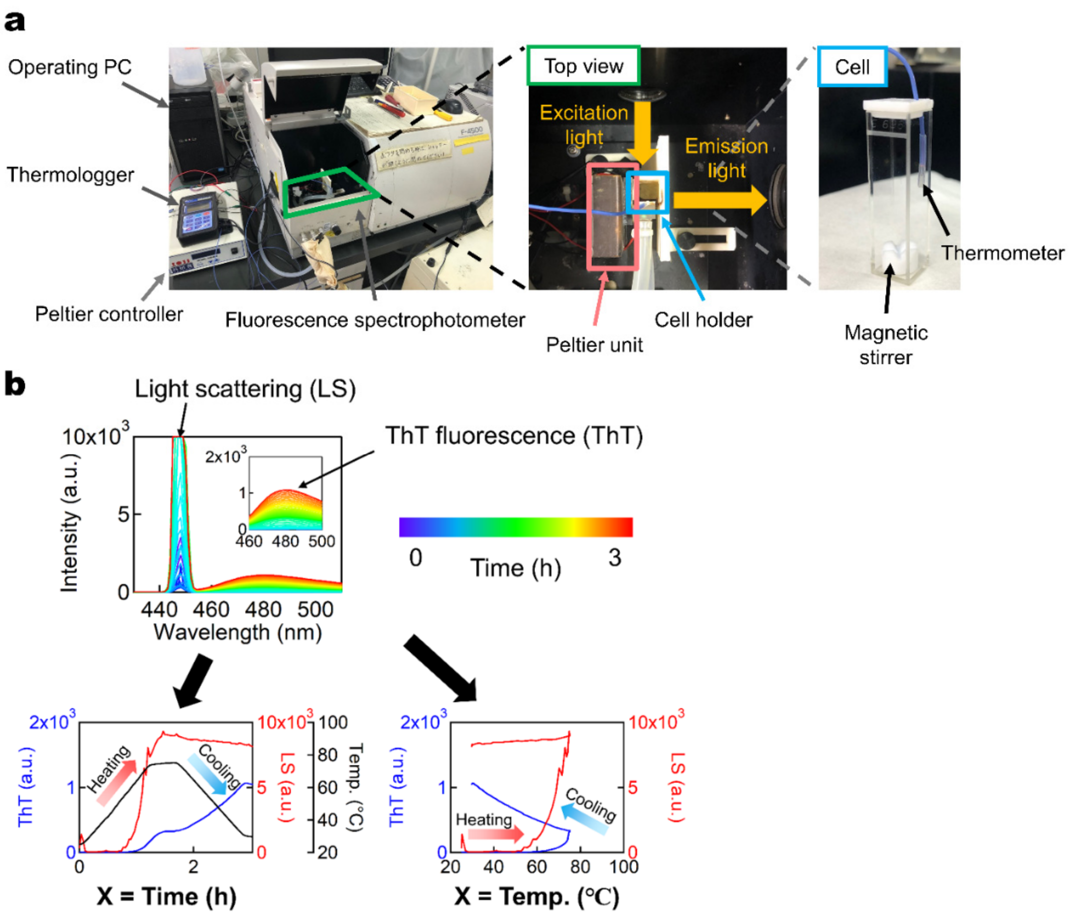

Supplementary Figure 1

Observation of heating-induced aggregation by simultaneous monitoring of light scattering and ThT fluorescence. a, Experimental set-up for the simultaneous monitoring of LS and ThT fluorescence under controlled heating and stirring. When heating was performed under stirring, a stirring speed of 800 rpm was employed using a magnetic stirring bar. The heating rate was controlled using a Peltier element (Nippon Tecmo Co., Ltd.) and the sample temperature was measured by a thermocouple (Anritsu Meter Co., Ltd.). **b,** Fluorescence spectra at different temperatures were measured repeatedly with an excitation wavelength of 445 nm at a heating rate of 1 °C/min. The spectra were converted to the dependences of ThT fluorescence at 485 nm and LS at 445 nm on incubation period or temperature. Similar measurements and analyses were carried out upon reducing the temperature after heating.

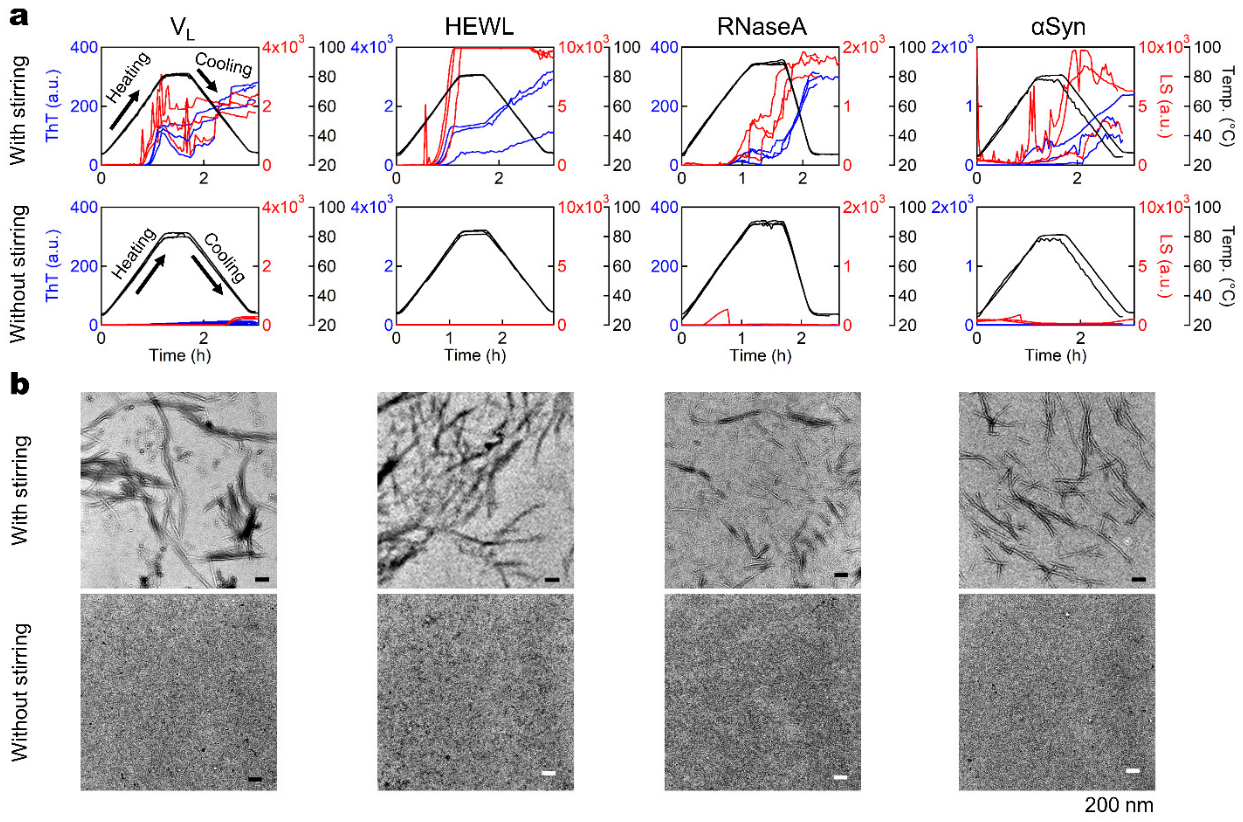

Supplementary Figure 2.

Heating- and agitation-dependent amyloid formation of type S proteins. a, The dependences of ThT fluorescence at 485 nm and LS at 445 nm on the incubation period at a heating rate of 1 °C/min for V_L, HEWL, RNaseA, and αSyn in the presence (upper) or absence (lower) of stirring. The intensities of ThT fluorescence and LS are indicated by blue and red lines, respectively. n = 3. **b,** TEM images of samples with and without stirring after the ThT assays. Scale bars; 200 nm.

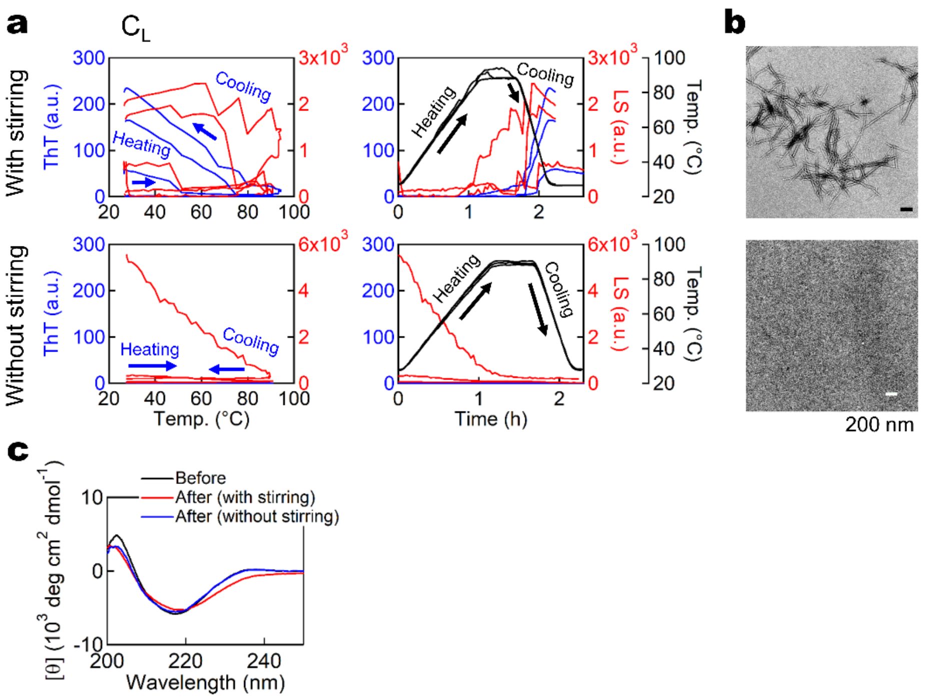

Supplementary Figure 3.

Heating- and agitation-dependent amyloid formation of C_L. a, The dependences of ThT fluorescence at 485 nm and LS at 445 nm on temperature (left) or the incubation period (right) at a heating rate of 1 °C/min in the presence (upper) or absence (lower) of stirring. The intensities of ThT fluorescence and LS are indicated by blue and red lines, respectively. *n* = 3. **b,** TEM images of samples with and without stirring after the ThT assays. Scale bars; 200 nm. C, CD spectra before and after the ThT assays.

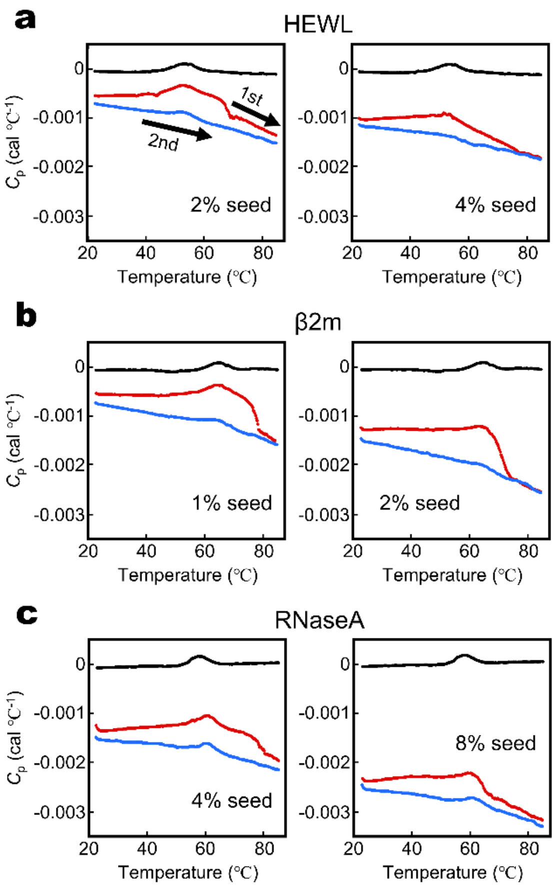

Supplementary Figure 4.

Seed-dependent amyloid formation of HEWL (a), β2m (b), and RNaseA (c) monitored by DSC. DSC measurements were performed in the absence (black) and presence (red) of seeds at the indicated seed concentrations. The lines represent the C_p values and blue lines represent the second heating in the presence of seeds. Solvent conditions were the same as in Table 1. The measurements for β2m followed Noji et al.[4], reproducing the previously reported results. In the absence of seeds, DSC showed a typical heat absorption peak coupled with protein unfolding (black lines)[5]. In the presence of seeds, DSC exhibited the downward shift of heat capacity coupled with protein unfolding (red lines), representing the heat of amyloid formation. The second heating in the presence of seeds (blue lines) show the heat capacity of amyloid fibrils because they were stable after the first heating up to 90 °C[6].

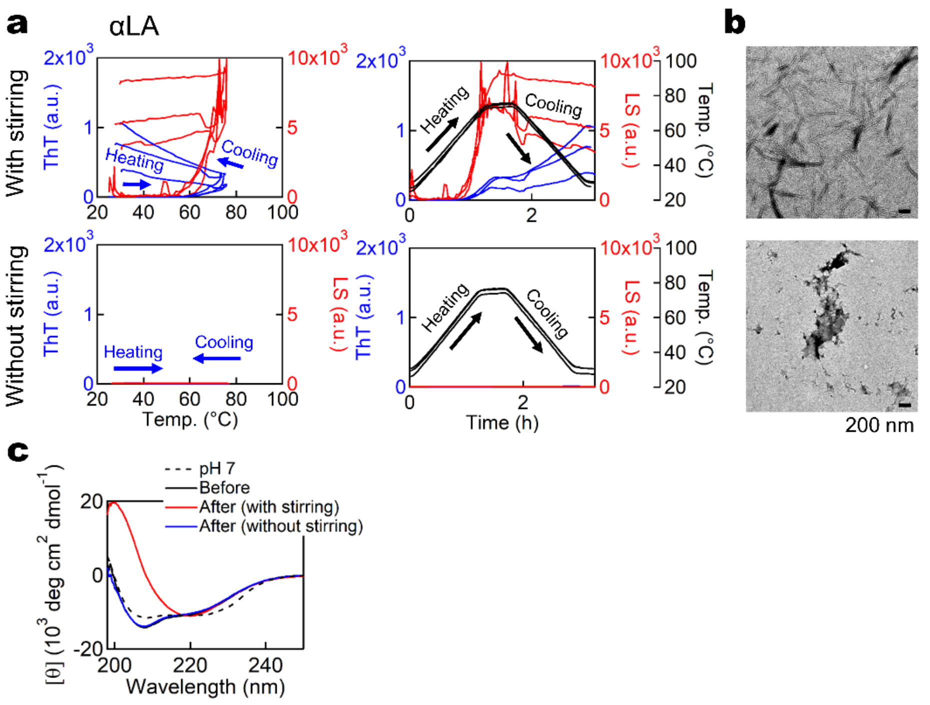

Supplementary Figure 5.

Heating- and agitation-dependent amyloid formation of αLA at pH 2.0 with 0.5 M NaCl.
a, The dependences of ThT fluorescence at 485 nm and LS at 445 nm on temperature (left) or the incubation period (right) at a heating rate of 1 °C/min in the presence (upper) or absence (lower) of stirring. The intensities of ThT fluorescence and LS are indicated by blue and red lines, respectively. n = 3. **b,** TEM images of samples with and without stirring after the ThT assays. Scale bars; 200 nm. **c**, CD spectra before and after the ThT assays. For comparison, the spectrum of the native state at pH 7 is also shown.

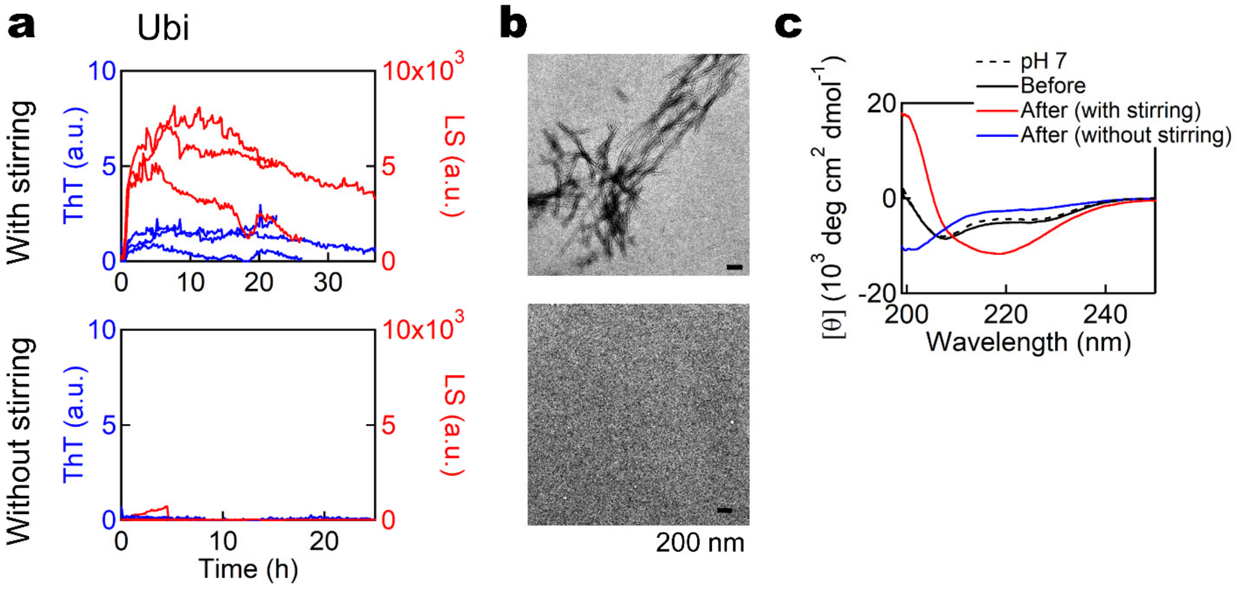

Supplementary Figure 6.

Heating- and agitation-dependent amyloid formation of ubiquitin at pH 2.0 with 0.5 M NaCl. **a,** The dependences of ThT fluorescence at 485 nm and LS at 445 nm on the incubation period at 90 °C in the presence (upper) or absence (lower) of stirring. The intensities of ThT fluorescence and LS are indicated by blue and red lines, respectively. $n = 3$. **b,** TEM images of samples with and without stirring after the ThT assays. Scale bars; 200 nm. **c**, CD spectra before and after the ThT assays. For comparison, the spectrum of the native state at pH 7 is also shown.

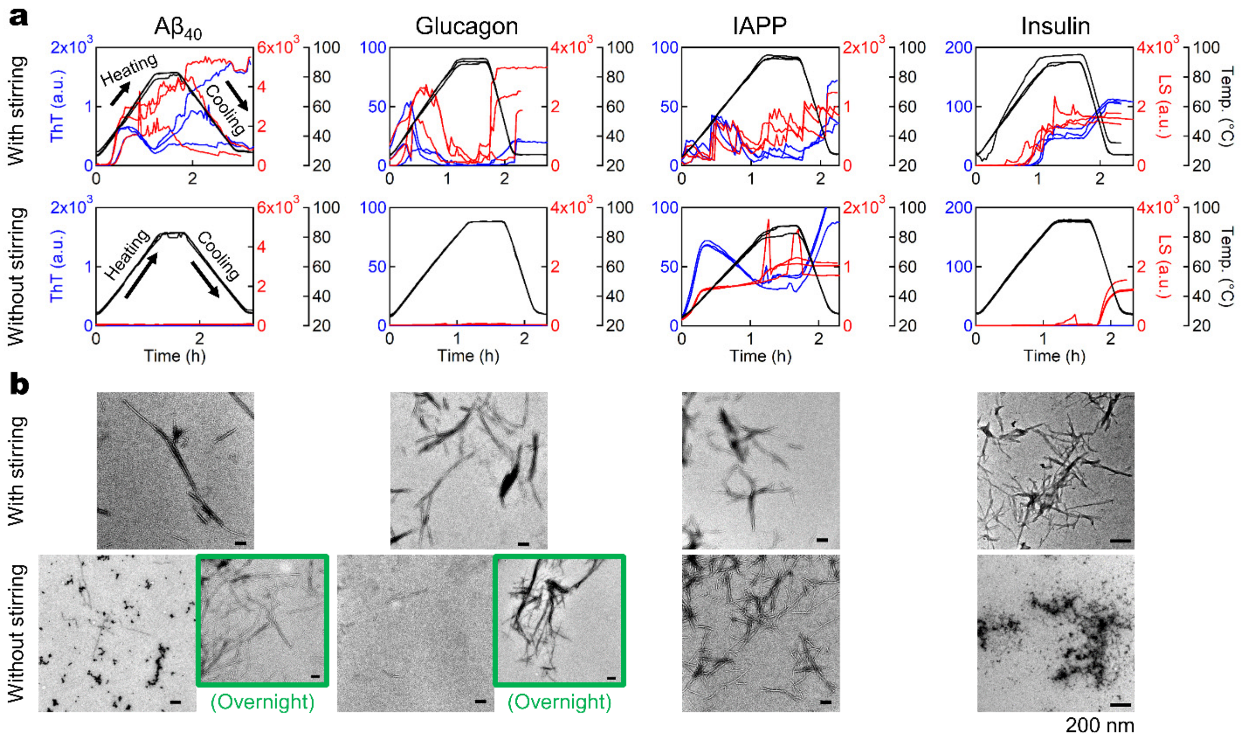

Supplementary Figure 7.

Heating- and agitation-dependent amyloid formation of type A proteins. a, The dependences of ThT fluorescence at 485 nm and LS at 445 nm on the incubation period at a heating rate of 1 °C/min for Aβ40, glucagon, IAPP, and insulin in the presence (upper) or absence (lower) of stirring. The intensities of ThT fluorescence and LS are indicated by blue and red lines, respectively. $n = 3$. **b,** TEM images of samples with and without stirring after the ThT assays. For Aβ40 and glucagon, TEM images after overnight incubation are shown because imaging depended on the time after the ThT assays. Scale bars; 200 nm.

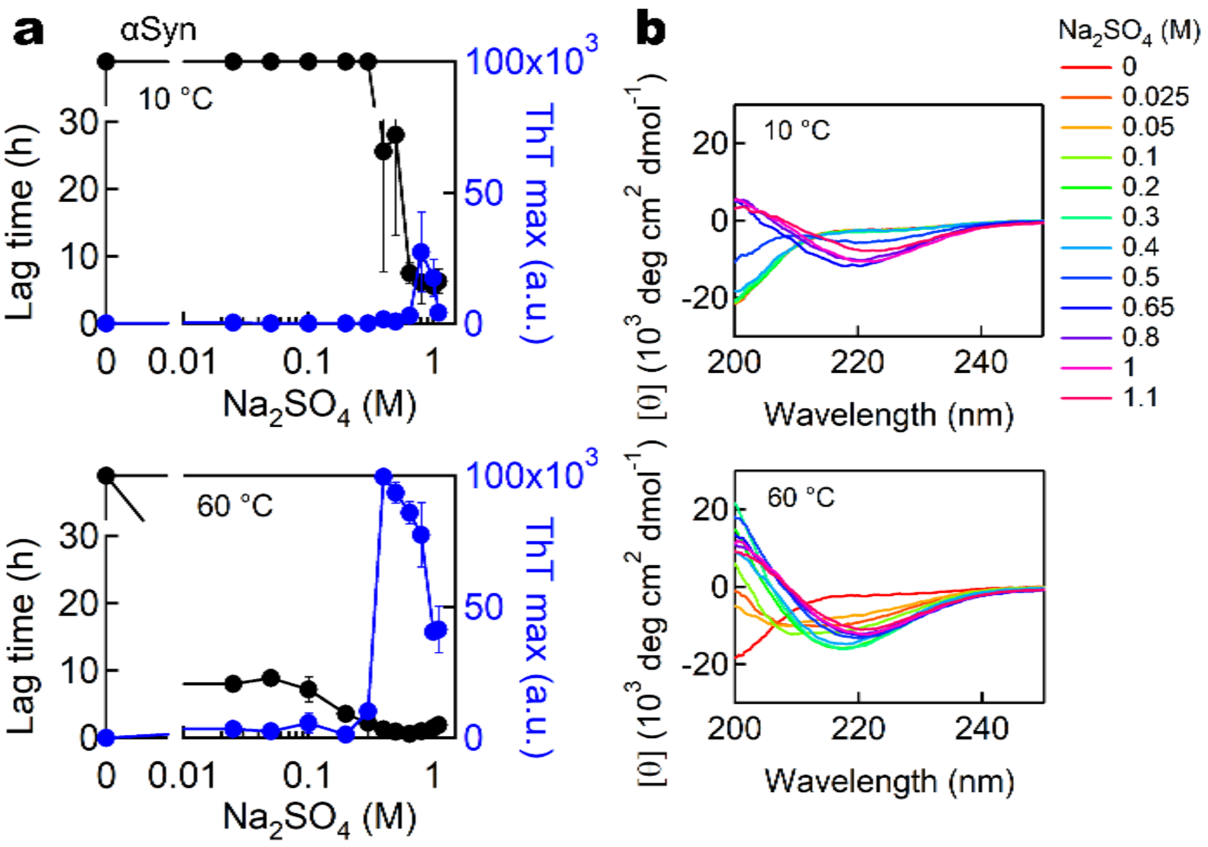

Supplementary Figure 8.

Heating- and agitation-dependent amyloid formation of αSyn. a, Dependence of 0.1-mg/mL αSyn amyloid formation on sodium sulfate concentrations (0–1.1 M) at 10 (upper) or 60 (lower) °C monitored by ThT assays (blue) and lag time (black). In these experiments, ultrasonic agitation was used instead of stirring. The experiments were performed using a fluorescence spectrophotometer with an attached ultrasonicator as previously described[7]. n = 5. **b,** CD spectra after ThT assays. The CD measurements were performed at 25 °C and the line representations for the spectra are described in the panels.

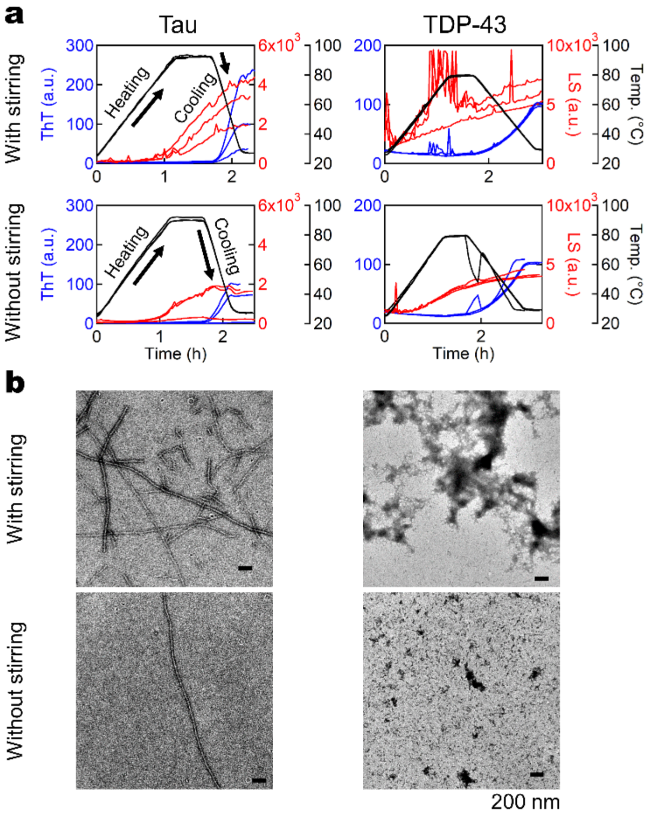

Supplementary Figure 9.

Heating- and agitation-dependent amyloid formation of type B proteins. a, The dependences of ThT fluorescence at 485 nm and LS at 445 nm on the incubation period at a heating rate of 1 °C/min for Tau and TDP-43 in the presence (upper) or absence (lower) of stirring. The intensities of ThT fluorescence and LS are indicated by blue and red lines, respectively. $n = 3$. **b,** TEM images of samples with and without stirring after the ThT assays. Scale bars; 200 nm.

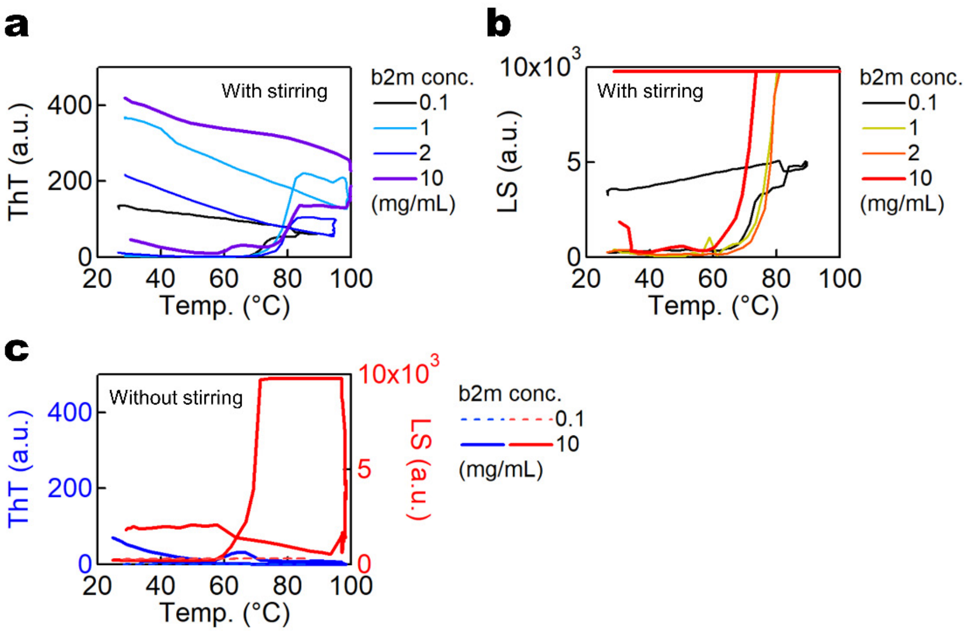

Supplementary Figure 10.

Effects of β2m concentration on the heating- and agitation-dependent amyloid formation of β2m. a, b The dependences of ThT fluorescence (**a, c**) at 485 nm and LS (**b, c**) at 445 nm on the temperature at a heating rate of 1 °C/min at various protein concentrations in the presence (**a, b**) or absence (**c**) of stirring.

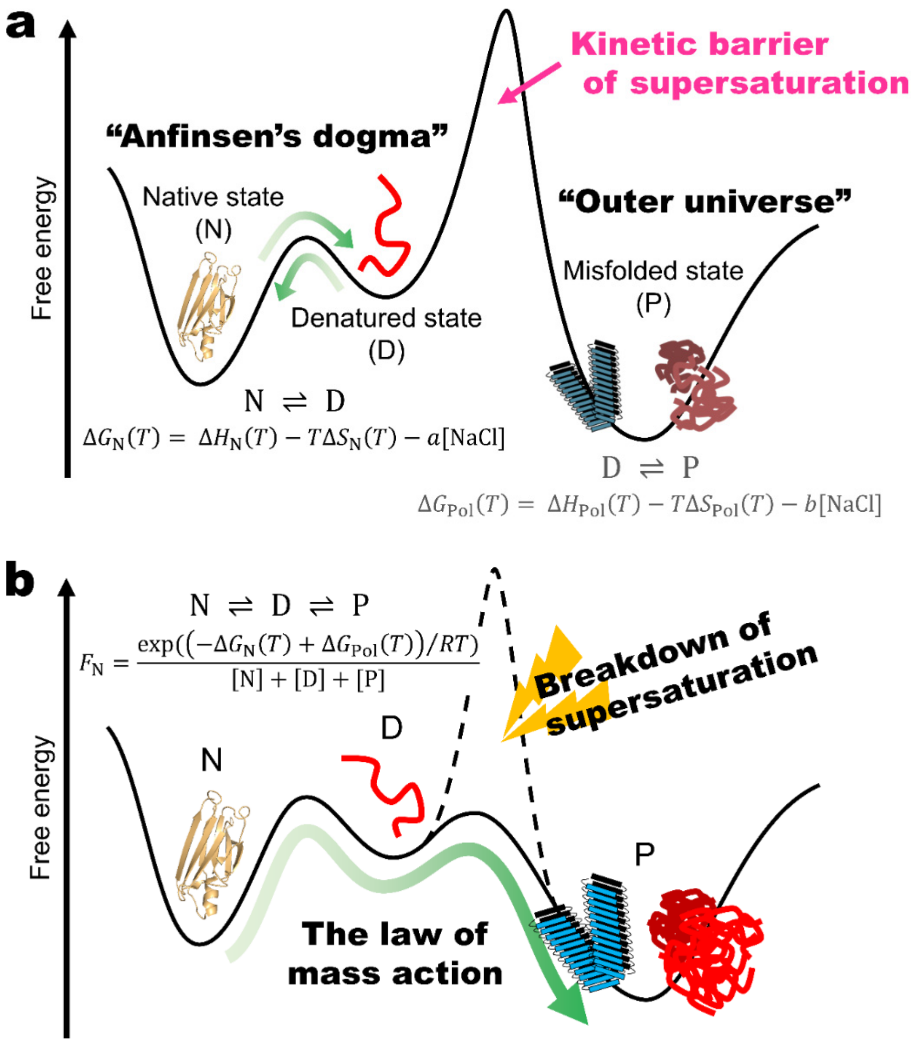

Supplementary Figure 11.

Schematic representations of free energies before (a) and after (b) the linkage of folding and misfolding transitions. (a) A two-state transition independent of protein concentration persists because the high free energy barrier of supersaturation and folding/unfolding transition between the native (N) and unfolded (D) states is consistent with Anfinsen's dogma. The structure of β2-microglobulin [PDB ID: 1JNJ] is shown for N. **(b)** Upon breaking supersaturation by agitation or seeding, the folding/unfolding transition and aggregation to polymeric states (P) enter an equilibrium, in which the solubility of D determines the overall equilibrium. When the concentration of the unfolded state is higher than its equilibrium solubility, this thermodynamic equilibrium after breaking supersaturation reduces the native concentration because of the law of mass action, thus destabilizing the native state.

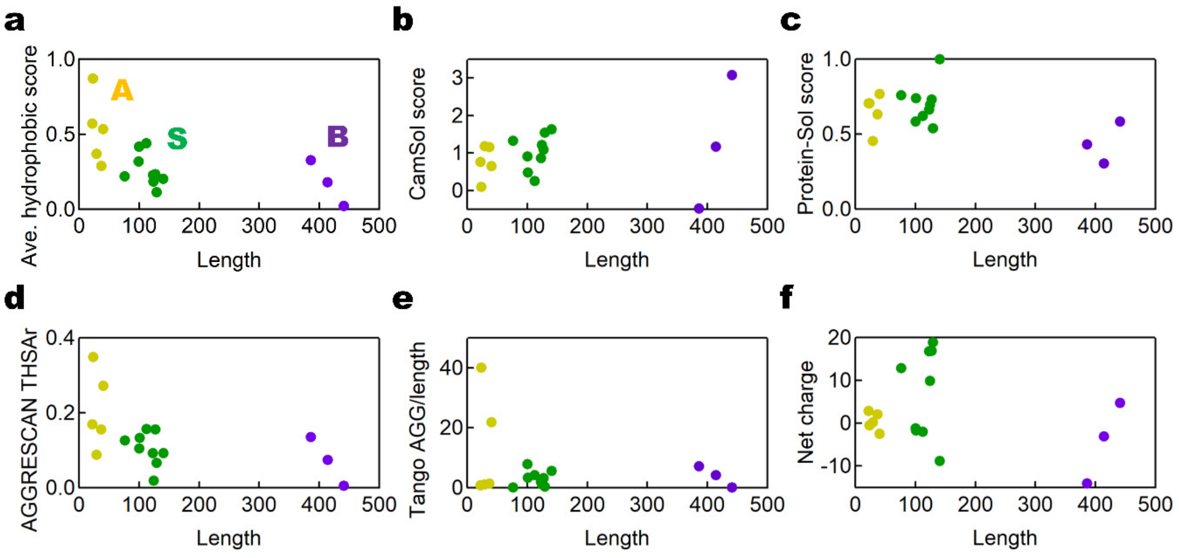

Supplementary Figure 12.

Proteins with distinct transition types (i.e., S, A and B types) plotted on the diagram of various factors (ordinate) against the number of amino acid residues (abscissa). Various factors are average hydrophobic (a), CamSol[1] (b), protein solubility (c), AGGRESCAN (d), and Tango AGG (e) scores as well as net charge (f). Their values are shown in **Supplementary Table 1.**

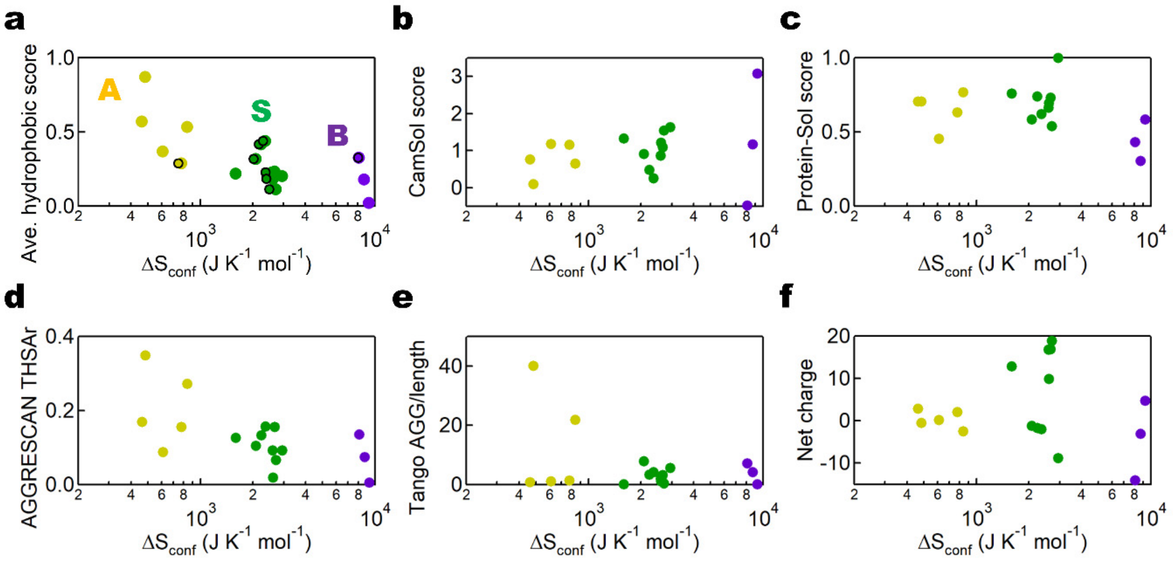

Supplementary Figure 13.

Proteins with distinct transition types (i.e., S, A and B types) plotted on the diagram of various factors (ordinate) against ΔSconf (abscissa). Various factors are average hydrophobic (a), CamSol[1] (b), protein solubility (c), AGGRESCAN (d), and Tango AGG (e) scores as well as net charge (f). Their values are shown in **Supplementary Table 1.** ΔSconf represents an increase upon denaturation of the main-chain with (points with frame) and without (points without frame) the contribution of disulfide bonds (Table 1).

Supplementary references

1 Sormanni, P., Aprile, F. A. & Vendruscolo, M. The CamSol method of rational design of protein mutants with enhanced solubility. *J Mol Biol* **427**, 478-490 (2015).

2 Hebditch, M., Carballo-Amador, M. A., Charonis, S., Curtis, R. & Warwicker, J. Protein-Sol: a web tool for predicting protein solubility from sequence. *Bioinformatics* **33**, 3098-3100 (2017).

3 Conchillo-Sole, O., de Groot, N. S., Aviles, F. X., Vendrell, J., Daura, X. & Ventura, S. AGGRESCAN: a server for the prediction and evaluation of "hot spots" of aggregation in polypeptides. *BMC Bioinformatics* **8**, 65 (2007).

4 Noji, M., Sasahara, K., Yamaguchi, K., So, M., Sakurai, K., Kardos, J., Naiki, H. & Goto, Y. Heating during agitation of β2-microglobulin reveals that supersaturation breakdown is required for amyloid fibril formation at neutral pH. *J Biol Chem* **294**, 15826-15835 (2019).

5 Makhatadze, G. I. & Privalov, P. L. Energetics of protein structure. *Adv Protein Chem* **47**, 307-425 (1995).

6 Sasahara, K., Yagi, H., Naiki, H. & Goto, Y. Heat-induced conversion of β2-microglobulin and hen egg-white lysozyme into amyloid fibrils. *J Mol Biol* **372**, 981-991 (2007).

7 Yoshimura, Y., Lin, Y., Yagi, H., Lee, Y. H., Kitayama, H., Sakurai, K., So, M., Ogi, H., Naiki, H. & Goto, Y. Distinguishing crystal-like amyloid fibrils and glass-like amorphous aggregates from their kinetics of formation. *Proc Natl Acad Sci U S A* **109**, 14446-14451 (2012).
