# Supplementary material for: Breakdown of supersaturation barrier links protein folding to amyloid formation
Source: Commun Biol. 2021 Jan 26;4:120. doi: 10.1038/s42003-020-01641-6 (PMC7838177; doi:10.1038/s42003-020-01641-6)
Supplement: Supplementary file 3 — Description of Additional Supplementary Files [file 42003_2020_1641_MOESM3_ESM.pdf]

Description of Additional Supplementary Files

File name: Supplementary Data 1

Description: The source data underlying Figs. 1a, b, 2a, c, e, d, 3a, b, 4a, b, and Supplementary Figs 2a, 3c, 4, 5a, c, 6a, c, 7a, 8a, b, 9a, 10.

File name: Supplementary Movie 1.

Description: Supersaturation- Final frontier in protein science. (a) Different conformational states of a monomeric globular protein with snake puzzle models of the native state (N), denatured or unfolded state (D), and polymeric amyloid state (P). (b) Relationship of folding and amyloid formation. (c) Protein folding, independent of protein concentration. At the midpoint of unfolding transition with a total number of monomers to be 10, protein concentration-independent unfolding/refolding equilibrium holds with [N] = 5 and [D] = 5, in agreement with Anfinsen's dogma. (d) Protein misfolding, dependent on protein concentration. High free energy barrier of supersaturation is broken by agitation or seeding, triggering the conversion of excess amount of unfolded monomers ([D] = 4) above solubility ([D] =1) to amyloid fibrils. (e) Establishment of the linked equilibrium of protein concentration-independent folding and protein concentration-dependent misfolding. [N] : [D] : [P] = 1 : 1 : 8. It is noted that amyloid formation apparently destabilizes the native state because of a mass action.
